# Comparison of the Essential Oil Composition of Selected *Impatiens* Species and Its Antioxidant Activities

**DOI:** 10.3390/molecules21091162

**Published:** 2016-09-01

**Authors:** Katarzyna Szewczyk, Danuta Kalemba, Łukasz Komsta, Renata Nowak

**Affiliations:** 1 Department of Pharmaceutical Botany, Medical University of Lublin, Chodźki 1 St., 20-093 Lublin, Poland; ren101d@gmail.com; 2 Institute of General Food Chemistry, Lodz University of Technology, Stefanowskiego 4/10 St., 90-924 Łódź, Poland; danuta.kalemba@p.lodz.pl; 3 Department of Medicinal Chemistry, Medical University of Lublin, Jaczewskiego 4, 20-090 Lublin, Poland; lukasz.komsta@umlub.pl

**Keywords:** *Impatiens*, Balsaminaceae, herb, root, essential oils, antioxidants, PCA

## Abstract

The present paper describes the chemical composition of the essential oils obtained by hydrodistillation from four *Impatiens* species, *Impatiens glandulifera* Royle, *I. parviflora* DC., *I. balsamina* L. and *I. noli-tangere* L. The GC and GC-MS methods resulted in identification of 226 volatile compounds comprising from 61.7%–88.2% of the total amount. The essential oils differed significantly in their composition. Fifteen compounds were shared among the essential oils of all investigated *Impatiens* species. The majority of these constituents was linalool (0.7%–15.1%), hexanal (0.2%–5.3%) and benzaldehyde (0.1%–10.2%). Moreover, the antioxidant activity of the essential oils was investigated using different methods. The chemical composition of the essential oils and its antioxidant evaluation are reported for the first time from the investigated taxon.

## 1. Introduction

The genus *Impatiens* L. (Balsaminaceae) includes about 850 species, which occur mainly in tropical and subtropical climate zones, in particular in parts of the Old World, such as tropical Africa, India, southern China and the southwestern part of Asia. Some species also occur in Japan, Europe, Russia and North America [1,2]. Three species, *Impatiens glandulifera* Royle (Himalayan balsam), *I. noli-tangere* L. (touch-me-not balsam) and *I. parviflora* DC. (small balsam), occur in Central Europe, and they are perennial plants that grow in riparian zones along rivers on humid soils and in wet woodlands [3,4]. *I. glandulifera* and *I. parviflora* are among the invasive plants originally native to Asia that are rapidly spreading across Europe. In Poland, these are two of the top 20 invasive alien plants [2,5]. As *I. parviflora* is an extremely invasive plant in Europe, its relation with other plants [6] and with soil yeast complexes was recently investigated [7]. Furthermore, the different extracts from *I. glandulifera*, *I. noli-tangere* and *I. parviflora* showed a strong allelopathic effect on the seed germination of *Leucosinapis alba* and *Brassica napus* [4].

Many groups of active compounds have been isolated from different species of the genus *Impatiens* L. Phytochemical studies conducted on various organs of *Impatiens* have revealed the presence of quinones, flavonoids, phenolic acids, leucocyanidins, anthocyanins, tannins, coumarins, saponins, phytosteroids, peptides, alkaloids and essential oils [8,9,10,11,12,13,14,15,16]. However, only one report is available concerning the volatile constituents of *Impatiens* species. In the n-hexane extract of *I. bicolor* growing in Pakistan, fatty acid methyl esters were the major compounds [9].

Because of the rich and varied composition, numerous studies have been conducted to investigate the feasibility of a medicinal use of members of *Impatiens*. Among the representatives of the genus *Impatiens* L., some species have been used since a very long time in Asian and American medicine. In traditional therapeutic systems, *I. balsamina* L. has been the most popular species. Depending on the type of ailment, the dried herb has been used in the form of compresses, directly on the skin, or as a tea prepared by pouring hot water on dried leaves [14]. Moreover, it has been applied in Chinese traditional medicine to treat rheumatism, against fractures, swelling, contusions, beriberi disease and for its anticancer properties [16,17,18]. It has been also used to alleviate parturient and puerperal pain [14]. *I. balsamina* flowers have been used as a remedy against the effects of snake bites [19]. *I. parviflora* has been used in the treatment of warts [20]. Flowers of *I. glandulifera* are used in Bach flower remedies, which causes sedation, relaxes and helps to balance the emotional state, and they are recommended for psychological problems and pain [21]. The rhizomes of *Impatiens pritzellii* Hook. f. var. *hupehensis* Hook. f. [22] and whole plant of *Impatiens textori* MIQ [23] have been also used in Chinese medicine. In our previous study, we confirmed that the extracts from species of *Impatiens*, especially *I. balfourii* Hook. f., *I. glandulifera* and *I. parviflora*, contained significant amounts of phenols and flavonoids and have interesting multidirectional biological activity, such as antimicrobial and antioxidant abilities [11].

Based on the significance of these plants from an ecological perspective and no available reports on the essential oils of *Impatiens* species, the aim of the present study was to investigate the essential oil composition of herb and root of the two most invasive in Poland *Impatiens* species, *I. glandulifera* Royle and *I. parviflora* DC. For comparison, herb oils of two other species, *I. balsamina* L. and *I. noli-tangere* L., were included. The antioxidant activity of the essential oils of these species was also evaluated.

## 2. Results and Discussion

### 2.1. Chemical Composition of Essential Oils from Impatiens L.

Six *Impatiens* essential oils were obtained by steam distillation from air-dried herbs and roots. All of them were yellowish and fragrant. The highest yield of essential oil (*w/w* relative to dry material weight) was observed for the herb of *I. parviflora* (0.24%) and *I. glandulifera* (0.22%), while for the other materials, the yield amounted to 0.10%–0.16%. The chemical composition was analyzed by the GC-MS method, which resulted in identification of 54–94 volatile compounds comprising from 61.7%–88.2% of the total volume in individual oils. In total of 226 compounds was identified. All identified compounds in *Impatiens* oils are listed in Table 1.

The essential oils differed significantly in their chemical composition. What is more, all investigated oils contained specific constituents that distinguished the essential oils from each other. However, some similarities in the qualitative composition can be observed.

Seventy six compounds were identified in the herb oil of *I. glandulifera*, representing 82.5% of the total oil. The oil was dominated by oxygenated monoterpenes (28.2%), and α-terpinyl acetate (16.6%) was the major constituent, followed by phellandral (3.8%). Phthalides were the most characteristic constituents of this oil: (*Z*)-ligustilide (11.0%) and (*Z*)-butylidenphthalide (8.5%) were accompanied by small amounts of their (*E*)-isomers and butylphthalide. This oil was the only one that contain pronounced amounts of monoterpene hydrocarbons (9.9% in total), and β-phellandrene (7.4%) was the main one in this group.

Root oil of *I. glandulifera* (94 compounds, 87.4%) had a totally different composition than herb oil. Three major groups of the constituent were aliphatic, mono- and sesquiterpene oxygenated compounds, each amounting to ca. 20%. The main component was sesquiterpene ketone vulgarone B (14.9%). Linalool (5.3%), borneol (4.9%) and bornyl acetate (4.3%) were the major monoterpenes. Another important constituent was pentadecanal (5.8%). The main feature of this oil was the presence of sixteen sesquiterpene hydrocarbons (12.7%), with β-barbatene (5.3%) being the major one. Eighteen constituents were identified in both herb and root oils of this species, e.g., hexanal, heptanal, nonanal, benzaldehyde, linalool, borneol and bornyl acetate, terpinene-4-ol, α-terpineol, β-ionone and its epoxide. It is worth mentioning that among the twelve sesquiterpene hydrocarbons ((5.1%) that were identified in the herb oil of this species, only one, δ-cadinene, was common for both oils. 

In the essential oil of *I. parviflora* herb, seventy compounds were identified amounting to 88.2%, and among them, (*E*)-hex-3-en-1-ol (16.8%), linalool (15.1%) and benzaldehyde (10.2%) were the most prominent. The number of identified components in the oil of *I. parviflora* roots was eighty nine (86.3% of the total oil), and the major compounds were citronellol (17.9%), geranial (12.8%) and linalool (4.9%). Despite significant differences in the content of major constituents, both herb and root oils contained the same aliphatic saturated and unsaturated alcohols, aldehydes and ketones C6–C16, which were their common distinctive features and constituted 42.8% and 26.1%, respectively. Isomeric heptadienals and octadienones that were identified in these oils were only rarely identified in the remaining investigated oils. 

The yield of essential oil obtained from *I. balsamina* herb was 0.1%. Eighty components were identified, representing 84.2% of this oil. The major constituent was hexahydrofarnesyl acetone (13.4%). Pronounced contents of ionones and damascones (15.8%), as well as fatty acids C6-C16 (9.5%) and alkanes (5.9%) were characteristic features of this oil. The main member of the first group was (*E*)-β-ionone (5.7%), and of the second group, dodecanoic acid (4.1%), ionones and damascones, which occur in a variety of essential oils, are degradation products of carotenoids and have the same C13 carbon skeleton, but differ in the site of oxygenation. (*E*)-β-Ionone and its epoxide were also found in other investigated oils, however in smaller amounts. Among 54 identified constituents, which accounted for 61.7% of the total essential oil from *I. noli-tangere* herb, the main compounds were (*Z*)-hex-3-enol (9.5%), linalool (6.5%) and benzaldehyde (4.7%).

Fifteen compounds were shared among the essential oils of all investigated *Impatiens* species. The majority of these constituents was linalool (0.7%–15.1%), hexanal (0.2%–5.3%) and benzaldehyde (0.1%–10.2%). Linalool is a naturally occurring monoterpene constituent found in more than 200 oils obtained from herbs, leaves, flowers and wood. This compound has many proven activities and is present in several remedies used in traditional medicine for sedative purposes. Moreover, linalool revealed antimicrobial, anti-inflammatory, antihyperalgesic and analgesic properties [24]. Chang and Shen investigated the cytotoxic activity of linalool. This study suggested good inhibitory effects against breast, colorectal and liver cancer cells [25].

According to the only available report on the composition of *Impatiens* volatiles, 42 components were characterized in the *n*-hexane extract of *I. bicolor* growing in Pakistan. The major ones were fatty acid methyl esters, such as *trans*-methyl 13-octadecenoate, methyl heptadecanoate, methyl octadecanoate, methyl docosanoate, methyl tetracosanoate, and methyl eicosanoate and aliphatic hydrocarbons [9]. 

Compounds of these two groups were identified in the essential oils of all investigated *Impatiens* species. However, their content was significantly lower. 

The invasive ability of some vigorous nonnative plants was thought to be associated with the competitive ability of the invasive species or a release from natural enemies. The allelopathic activity of invasive species also has recently been reported as a significant factor that negatively influences species biodiversity and ecosystem succession [26]. Among the allelochemicals, essential oils and their individual components belong to the most investigated [27]. Oxygenated monoterpenes were proven to possess high phytotoxic activity that inhibits the seed germination and seedling growth of many plants [28]. Terpinene-4-ol, which is a minor constituent of each investigated oil, appeared to be the most active of the 47 monoterpenes against *Lactuca sativa*, and the linalool, citronellol and geranial, major constituents of *I. glandulifera* and/or *I. parviflora*, revealed a pronounced phytotoxic effect [29].

### 2.2. Chemometric Analysis

**Figure 1:**
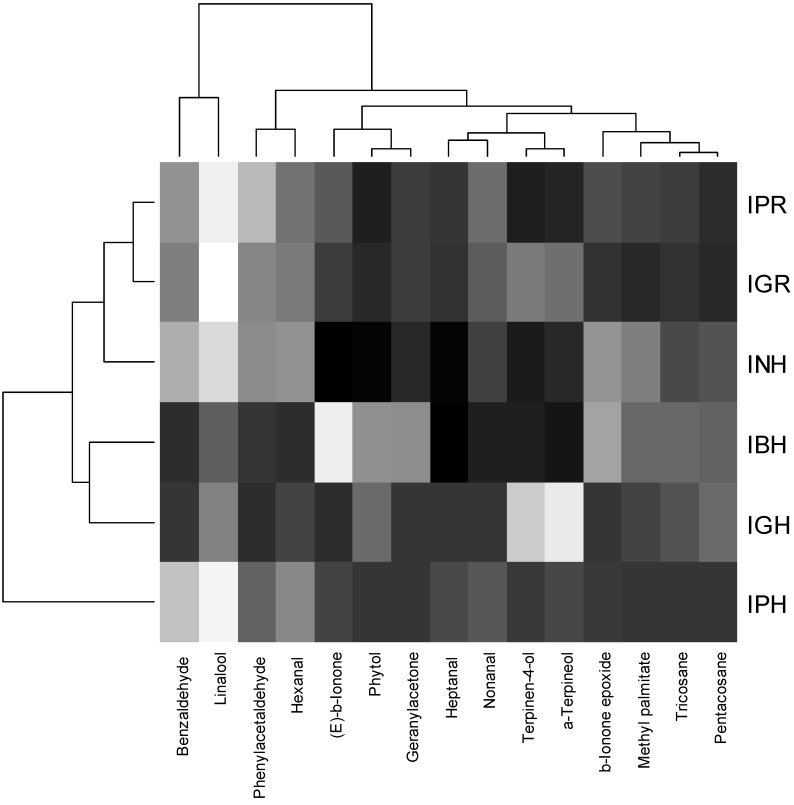
The heatmap analysis of the essential oil constituents. IBH, *I. balsamina* herb; IGH, *I. glandulifera* herb; IGR, *I. glandulifera* roots; INH, *I. noli-tangere* herb; IPH, *I. parviflora* herb; IPR, *I. parviflora* roots.

The main constituents common for all tested essential oils (hexanal, heptanal, benzaldehyde, phenylacetaldehyde, nonanal, linalool, terpinen-4-ol, α-terpineol, geranylacetone, β-ionone epoxide, (*E*)-β-ionone, methyl palmitate, phytol, tricosane, pentacosane) were compared with hierarchical cluster analysis with Euclidean distance as the similarity measure. The so-called “heatmap” with corresponding dendrograms is presented in Figure 1. Benzaldehyde and linalool form distinct cluster with different values than the rest of the analyzed main constituents. However, Euclidean distance does not uncover any distinct cluster among plant material samples, besides a distinct difference of *I. parviflora* herb (IPH) compared to the other samples.

**Figure 2:**
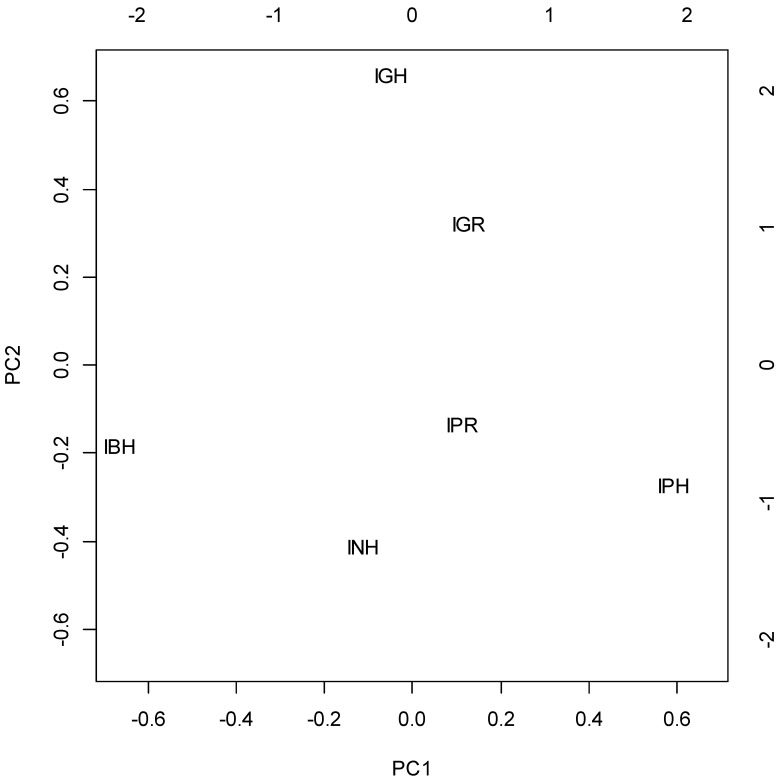
Scores of scaled principal component analysis: a comparison of the correlations between constituents among investigated materials. IBH, *I. balsamina* herb; IGH, *I. glandulifera* herb; IGR, *I. glandulifera* roots; INH, *I. noli-tangere* herb; IPH, *I. parviflora* herb; IPR, *I. parviflora* roots.

**Figure 3:**
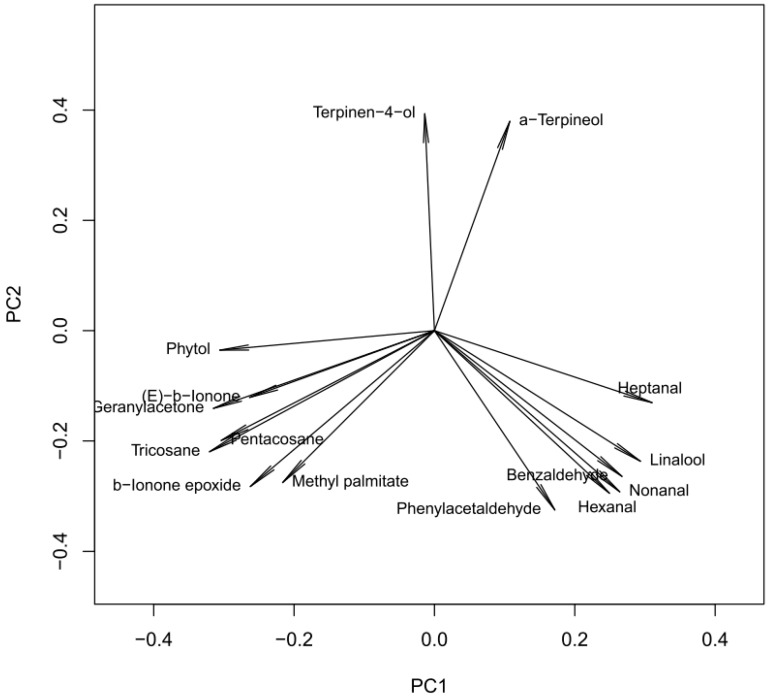
Loading vectors of scaled principal component analysis presented in Figure 2.

To the compare correlation between constituents (regardless of the absolute concentrations), scaled principal component analysis was carried out. Forty eight-point-four percent and 32.4% of variance was explained by the first two PCs, respectively (Figure 2). Analyzing the loading vectors (Figure 3), it can be concluded that the compounds form three intercorrelated groups:

1. Terpinen-4-ol and α-terpineol, explained mainly with PC2 and weakly (reversely) correlated with other compounds;
2. Hexanal, nonanal, linalool, heptanal and benzaldehyde, located mainly in PC1, intercorrelated and reversely correlated with Group 3;
3. Geranyl-acetone, β-ionone-epoxide, methyl-palmitate, phytol, tricosane and pentacosane, explained by PC1 and negatively correlated with Group 2.

*I. glandulifera* roots (IGR) and *I. glandulifera* herb (IGH) contain the high concentration of group (1), whereas other material samples have smaller concentrations of them, and the differences arelocated mainly along the PC1 axis.

### 2.3. Antioxidant Activity

In the present study, the antioxidant activities of the essential oils from herb and roots of Impatiens species were determined using two different methods. The free radical scavenging activity of essential oils was evaluated by the DPPH method in comparison with that of ascorbic acid, at different concentrations. Radical scavenging activity was expressed as the amount of antioxidants necessary to decrease the initial DPPH absorbance by 50% (EC_50_). The highest antiradical activity was detected for herb oils of *I. glandulifera* (3.96 ± 0.03 µg/mL) and *I. noli-tangere* (4.76 ± 0.05 µg/mL), whereas the lowest was detected for herb oil of *I. balsamina* (Table 2). The EC_50_ value of ascorbic acids to scavenge hydroxyl radicals was 2.05 ± 0.01 µg/mL. Our results are comparable to those obtained by Nisar and co-authors for the hexane extract of *I. bicolor*. The EC_50_ values obtained for different fractions ranged from 23.22 ± 0.75 µg/mL–59.00 ± 2.01 µg/mL, while the value for ascorbic acid was 7.80 ± 0.14 µg/mL [9]. 

The inhibition of linoleic acid peroxidation revealed low capacities of the essential oils of *Impatiens* species in comparison to the DPPH test (Table 2). The most active essential oils from herb and roots of *I. glandulifera* showed even up to six-times higher IC_50_ values than the lipophilic antioxidant BHT.

## 3. Experimental Section

### 3.1. Reagents and Materials

Aerial parts and roots of plants were collected during July–September 2014. *I. balsamina* L. (No. IB-0714) was collected in the Maria Curie-Skłodowska University (UMCS) Botanical Garden, which is a part of Maria Curie Sklodowska University in Lublin, Poland, at an altitude of 197 m a.m.s.l. (coordinates N 51°08’41’’; E 22°18’17’’). *I. glandulifera* Royle (No. IG-0814) and *I. noli-tangere* L. (No. INT-0914) were gathered in Józefów near Biłgoraj (Poland) at an altitude of 240 m a.m.s.l. (coordinates N 50°29’06’’; E 23°02’12’’ and N 52°57’58’’; E 23°04’46’’ respectively). *I. parviflora* DC. (No. IP-0914) was collected in Motycz Leśny near Lublin (Poland) at an altitude of 180 m a.m.s.l. (coordinates N 51°14’57’’; E 22°20’36’’). Voucher specimens were deposited in the Department of Pharmaceutical Botany, Faculty of Pharmacy, Medical University of Lublin. Plants were identified by Prof. Tadeusz Krzaczek.

All chemical reagents used in the experiment were purchased from various commercial suppliers and were of the highest purity available. 2,2-diphenyl-1-picrylhydrazyl (DPPH), ascorbic acid, 2,6-di-*tert*-butyl-4-methylphenol (BHT) and linoleic acid (LA) were purchased from Sigma-Aldrich (St. Louis, MO, USA).

### 3.2. Isolation Procedure

The essential oils (EOs) of 100 g of dried herb or roots of *Impatiens* species were obtained by hydrodistillation for 3 h using the Clevenger-type apparatus, according to European Pharmacopoeia 5.0. Next, the EOs were trapped in 2 mL of freshly-rectified diethyl ether. After distillation, the organic layers were collected and dried over anhydrous magnesium sulfate. After filtration and additional washing of diethyl ether, the solvent was evaporated at room temperature, and the residues were weighed. The oil yields were calculated on the basis of the dry weight of plant material and according to the formula [30]:

EO (%) = W1/W2 × 100(1)

where W1 is the net weight of oils (g) and W2 is the total weight of dry samples (g).

### 3.3. GC-MS Analysis

The chemical composition of the essential oils was analyzed using GC-MS on a Trace GC Ultra apparatus (Thermo Electron Corporation, Milan, Italy) with FID and the MS DSQ II detector after dilution in diethyl ether (10 μL in 1 mL). A simultaneous GC-FID and MS analysis was performed using an MS-FID splitter (SGE, Analytical Science, Austin, TX, USA). Operating conditions: apolar capillary column Rtx-1ms (Restek), 60 m × 0.25 mm i.d., film thickness 0.25 μm; temperature program, 50–310 °C at 2 °C/min; SSL (splitless) injector temperature 280 °C; FID temperature 300 °C; split ratio 1:20; carrier gas helium at a regular pressure 300 kPa. Mass spectra were acquired over the mass range of 30–400 Da, ionization voltage 70 eV; ion source temperature 200 °C. Identification of the components was based on a comparison of their mass spectra and relative retention indices with data stored in computer libraries NIST 98.1, Wiley 8th Ed. and MassFinder 4.1. Retention indices (RI, apolar column) were determined with relation to a homologous series of alkanes (C8–C26) under the same conditions with linear interpolation. Percentages were calculated from the FID response without the use of correction factors.

### 3.4. Free Radical Scavenging Activity

To determine the antioxidant activity of essential oils from *Impatiens* species, the method based on the reduction of the methanolic solution of colored free radical DPPH· was used. The changes in color from deep-violet to light-yellow were measured at 515 nm in a UV/visible light spectrophotometer (Thermo Evolution 300, Madison, WI, USA). Radical scavenging activity was measured according to the Brand-Williams et al. [31] method with the use of six dilutions of the analytes in methanol. The activity of ascorbic acid was evaluated for comparison. Antioxidant activity was expressed as EC_50_ (efficient concentration): the amount of dry extract (µg of DW) needed to obtain 50% activity per 1.0 mL of the initial solution.

### 3.5. Inhibition of Linoleic Acid Peroxidation

The antioxidant activity was also determined as the degree of inhibition on the hemoglobin-catalyzed peroxidation of linoleic acid according to the method described in previous studies [32,33] with a slight modification. The hydroxyperoxide formed was assayed according to the ferric thiocyanate method with mixing with 0.02 M FeCl_2_ followed by 30% ammonium thiocyanate. The absorbance of the sample (A_s_) was measured at 480 nm. The absorbance of blank (A_0_) was obtained without hemoglobin to the reaction mixture; the absorbance of the control (A_100_) was determined without the sample added to the mixture. Thus, the antioxidative activity of the sample was calculated according to the formula:

AA [%] = [(1 − (A_s_ − A_0_) / (A_100_ − A_0_)] × 100 (2)

Antioxidant activity was expressed as IC_50_ (inhibition concentration): the amount of antioxidant needed to decrease the linoleic acid peroxidation by 50%. 

### 3.6. Data Analysis

All measurements were performed at least in triplicate and expressed as the means ± standard deviations (±SD). Statistical significance was estimated through Tukey’s test for the data obtained from three independent samples of each essential oil in three parallel experiments (*n* = 9). Besides the classical pairwise correlation check, we applied the scaled principal component analysis. Statistical tests were performed using Statistica 6.0 software (Stat-Soft, Inc., Tulsa, OK, USA).

## 4. Conclusions

It is well understood that invasive species produce specific compounds affecting native plants occurring in the same habitat. This phenomenon is known as allelopathy. Identification of chemical constituents produced and emitted by invasive species helps to understand their impact on the local environment. 

Taking into account the chemical composition of *I. glandulifera* and *I. parviflora* essential oils and previous data on the allelopathic activity of monoterpenoids, it seemed possible that the emission of monoterpenes by herb and root of these two *Impatiens* species plays a role in their invasive ability. However, this hypothesis needs further research.

## Figures and Tables

**Table 1:** Composition of the essential oils of four *Impatiens* species. IGH, *I. glandulifera* herb; IGR, *I. glandulifera* roots; IPH, *I. parviflora* herb; IPR, *I. parviflora* roots; IBH, *I. balsamina* herb; INH, *I. noli-tangere* herb; RI_exp_, experimental retention index; RI_lit_, literature retention index; t, trace (percentage value less than 0.05%).

No.	Constituent	RI_exp_	RI_lit_	IGH	IGR	IPH	IPR	IBH	INH	Class of Compound
				Content (%)						
1	Hexanal	776	771	0.2	1.7	**5.3**	1.6	0.7	3.6	AO
2	Furfural	803	795	-	0.8	0.7	1.4	-	-	O
3	(*E*)-Hex-2-enal	826	822	-	0.3	2.1	1.4	2.9	-	AO
4	(*E*)-Hex-3-en-1-ol	838	838	0.6	-	**16.8**	0.3	0.7	-	AO
5	(*Z*)-Hex-3-en-1-ol	848	851	0.2	-	-	-	0.2	**9.5**	AO
6	Hexanol	852	856	-	1.7	**4.9**	0.3	-	-	AO
7	Heptan-2-one	868	871	-	0.2	0.2	0.1	-	0.2	AO
8	Heptanal	878	882	0.1	0.4	1.2	0.5	0.1	0.1	AO
9	2-Butoxyethanol	888	888	-	0.2	0.1	0.6	-	-	AO
10	Heptan-3-ol	893	884	-	0.1	-	-	-	-	AO
11	Butyraldehyde diethyl acetal	896	880	2.4	-	-	-	-	-	AO
12	Nonane	900	900	-	-	-	0.1	-	-	AH
13	1-Butoxypropan-2-ol	926	936	-	0.4	-		-	-	AO
14	Benzaldehyde	929	928	0.1	1.8	**10.2**	2.3	0.7	**4.7**	Ar
15	Dimethyl trisulfide	942	942	-	0.4	-	-	-	-	O
16	Benzonitrile	945	940	-	0.5	-	-	-	-	Ar
17	Isovaleraldehyde diethyl acetal	946	930	0.7	-	-	-	-	-	AO
18	Hept-1-en-3-one	954	956	-	0.1	-	-	-	-	AO
19	Heptanol	956	952	-	0.3	0.9	0.3	-	-	AO
20	Octane-2,3-dione	961	959	-	0.3	0.4	0.5	-	-	AO
21	Oct-1-en-3-ol	964	962	-	1.3	0.9	0.7	0.3	-	AO
22	6-Methylhept-5-en-2-one	964	962	-	-	0.3	-	-	-	AO
23	Octan-3-one	965	963	-	1.2	-	-	-	-	AO
24	β-Pinene	969	970	-	0.1	-	-	-	-	MH
25	(*E,E*)-Hepta-2,4-dienal	969	967	-	-	0.6	0.2	-	-	AO
26	2-Pentylfurane	979	981	-	0.3	1.2	0.8	0.4	1.4	O
27	Octanal	980	982	-	0.3	-	-	-	-	AO
28	Hexanoic acid	981	973	-	-	-	-	0.6	-	AO
29	Hepta-2,4-dienal (isomer)	981	-	-	-	4.2	1.2	-	1.2	AO
30	(*E*)-2-(Pent-2-enyl)furan	986	984	-	-	0.5	0.2	t	-	O
31	α-Phellandrene	997	997	0.5	-	-	-	-	-	MH
32	Decane	1000	1000	-	-	-	0.1	-	-	AH
33	Phenylacetaldehyde	1009	1012	t	2.0	2.7	3.3	0.8	3.4	Ar
34	α-Terpinene	1009	1013	0.2	-	-	-	-	-	MH
35	Salicylaldehyde	1011	1013	-	1.0	0.1	0.4	-	-	Ar
36	*p*-Cymene	1012	1015	0.7	-	-	-	-	-	MH
37	2,2,6-Trimethylcyclohexanone	1013	1013	-	-	t	-	t	-	O
38	2-Ethylhexan-1-ol	1015	1011	-	0.1	0.3	0.5	0.1	-	AO
39	β-Phellandrene	1022	1023	**7.4**	-	-	-	-	-	MH
40	Limonene	1025	1025	0.3	-	-	-	-	-	MH
41	(*Z*)-β-Ocimene	1028	1029	0.6	-	-	-	-	-	MH
42	(*E*)-Oct-2-enal	1032	1034	-	0.1	0.2	0.3	-	0.2	AO
43	Acetophenone	1033	1030	-	0.2	-	0.2	-	0.1	Ar
44	2,6,6-Trimethylcyclohex-2-enone	1037	1045	-	-	0.1	-	-	-	O
45	Octa-3,5-dien-2-one (isomer)	1043	-	-	-	0.3	0.4	-	-	AO
46	γ-Terpinene	1048	1059	0.1	t	-	-	-	0.1	MH
47	(*E*)-Oct-2-en-1-ol	1052	1052	-	0.2	0.6	0.1	-	-	AO
48	Octanol	1055	1054	0.2	-	-	-	0.1	-	AO
49	*trans*-Linalool oxide (F)	1058	1058	-	0.1	2.3	0.4	-	1.0	MO
50	Guaiacol	1061	1059	0.1	-	0.1	0.1	-	-	Ar
51	(*E*,*E*)-Octa-3,5-dien-2-one	1065	1063	-	-	0.4	0.4	0.1	-	AO
52	Nonan-2-one	1070	1072	-	-	-	0.1	-	-	AO
53	*cis*-Linalool oxide (F)	1072	1072	t	-	1.0	0.1	-	0.3	MO
54	6-Methylhepta-3,5-dien-2-one	1077	1075	-	-	0.1	0.3	-	-	AO
55	Terpinolene	1079	1082	0.1	-	-	-	-	-	MH
56	Nonanal	1083	1086	0.1	1.1	1.9	1.5	0.5	1.2	AO
57	Linalool	1086	1087	0.7	**5.3**	**15.1**	**4.9**	1.6	**6.5**	MO
58	Isophorone	1092	1094	-	-	0.1	-	t	-	MO
59	*cis*-Rose oxide	1096	1096	-	-	0.2	0.5	-	0.1	MO
60	α-Campholenal	1104	1105	t	-	-	-	-	-	MO
61	Oxoisophorone	1105	1005	-	-	t	0.3	t	0.2	MO
62	(*E*)-But-1-enylbenzene	1107	1110	0.5	-	-	-	-	-	Ar
63	*trans*-Rose oxide	1113	1114	-	-	0.1	0.3	-	0.1	MO
64	*trans*-Pinocarveol	1122	1126	-	0.3	-	-	-	-	MO
65	*trans*-*p*-Menth-2-en-1-ol	1123	1123	-	0.7	-	-	-	-	MO
66	Citronellal	1126	1129	-	-	0.4	0.5	-	-	MO
67	*o*-Acetylphenol	1129	1135	-	-	-	0.3	-	-	Ar
68	Hexyl isobutyrate	1133	1132	0.1	-	-	-	-	-	AO
69	4-Vinylanisol	1134	1134	-	-	-	-	0.2	-	Ar
70	(*E*)-Non-2-enal	1134	1139	-	-	-	0.5		-	AO
71	(*E*,*E*)-Nona-3,6-dien-1-ol	1136	1145	-	-	-	-	0.2	-	AO
72	Pinocarvone	1137	1137	-	0.1	-	-	-	-	MO
73	Pentylbenzene	1143	1150	0.3	-	-	-	-	-	Ar
74	Borneol	1149	1150	0.1	**4.9**	-	-	-	-	MO
75	*cis*- or *trans*-Linalool oxide (P)	1153	1148	-	-	0.1	-	-	-	MO
76	Benzoic acid	1156	1157	-	-	-	-	2.0	-	Ar
77	*p*-Cymen-9-ol	1156	1157	-	0.4	-	-	-	-	MO
78	Nonanol	1157	1149	-	-	-	0.9	-	-	AO
79	Cryptone	1157	1160	**5.7**	-	-	-	-	-	O
80	Terpinen-4-ol	1160	1164	1.5	1.7	0.3	0.2	0.5	0.5	MO
81	Octanoic acid	1164	1160	-	-	-	-	0.1	-	AO
82	*p*-Cymen-8-ol	1166	1169	-	-	-	0.3	0.5	-	MO
83	Methyl salicylate	1168	1171	-	-	-	-	0.1	-	Ar
84	Myrtenal	1168	1172	-	0.3	-	-	-	-	MO
85	α-Terpineol	1174	1176	1.9	1.5	1.0	0.3	0.4	0.7	MO
86	Safranal	1179	1182	-	-	0.3	t	0.5	t	MO
87	*cis*-Piperitol	1181	1181	0.1	-	-	-	-	-	MO
88	Myrtenol	1181	1184	-	0.6	-	**4.2**	1.2	-	MO
89	Decanal	1184	1184	-	0.8	0.1	1.6	0.4	-	AO
90	(*E*,*E*)-Nona-2,4-dienal	1186	1188	-	-	0.1	-	-	-	AO
91	Benzothiazole	1188	1186	-	0.1	-	-	-	-	O
92	3,5,5-Trimethyl-4-methylenecyclohex-2-enone	1190	1200	-	-	0.3	-	-	-	O
93	*trans*-Piperitol	1191	1193	0.3	-	-	-	-	-	MO
94	Octyl acetate	1193	1191	0.9	-	-	-	-	-	AO
95	Carvotanacetol	1193	1195	-	-	-	-	0.2	-	MO
96	β-Cyclocitral	1195	1195	-	0.1	0.6	0.1	0.5	0.4	MO
97	*trans*-Carveol	1199	1200	0.1	-	-	-	-	-	MO
98	*p*-Isopropylbenzaldehyde	1212	1214	0.9	-	-	-	-	-	Ar
99	Citronellol	1214	1213	-	-	2.5	**17.9**	-	1.9	MO
100	Carvone	1215	1214	0.6	-	-	-	-	-	MO
101	Piperitone	1226	1226	0.2	-	-	-	-	-	MO
102	(2,6,6-Trimethylcyclohex-1-en-1-yl)acetaldehyde	1235	1236	-	-	-	-	0.4	-	O
103	2-Phenylbut-2-enal	1235	1237	-	0.2	-	-	-	-	Ar
104	Geranial	1240	1235	-	-	0.7	**12.8**	-	-	MO
105	Phellandral	1251	1250	3.8	-	-	-	-	-	MO
106	4-Ethylguaiacol	1253	1257	0.2	-	-	-	-	-	Ar
107	Terpinen-7-al	1257	1257	0.3	-	-	-	-	-	MO
108	Nonanoic acid	1258	1263	-	-	-	-	0.2	-	AO
109	*p*-Cymen-7-ol	1266	1266	0.9	-	-	-	-	-	MO
110	Bornyl acetate	1268	1270	0.3	**4.3**	-	-	-	-	MO
111	Thymol	1271	1267	-	-	-	-	-	0.2	MO
112	Undecan-2-one	1273	1274	-	0.3	-	0.4	-	-	AO
113	Carvacrol	1280	1278	0.2	-	-	-	-	-	MO
114	4-Vinylguaiacol	1284	1282	-	-	0.3	1.2	1.7	-	Ar
115	Undecanal	1286	1286	-	0.5	-	0.6	-	2.1	AO
116	(*E*,*E*)-Deca-2,4-dienal	1289	1288	-	-	0.1	0.2	0.1	0.2	AO
117	Theaspirane A	1290	1293	-	-	0.1	-	-	-	O13
118	Theaspirane B	1304	1304	-	-	0.1	-	-	-	O13
119	Eugenol	1329	1331	-	-	1.0	-	0.1	1.1	Ar
120	1,1,6-Trimethyl-1,2-dihydronaphthalene	1335	1336	-	-	-	-	0.1	-	Ar
121	α-Terpinyl acetate	1336	1335	**16.6**	-	-	-	-	-	MO
122	(*E*)-Undec-2-enal	1339	1341	-	-	0.2	1.6	-	-	AO
123	Neryl acetate	1341	1342	-	-	-	-	-	0.2	MO
124	Decanoic acid	1352	1350	-	-	-	-	0.3	-	AO
125	α-Longipinene	1354	1360	-	0.3	-	-	-	-	SH
126	Geranyl acetate	1359	1362	0.6	-	-	-	-	-	MO
127	(*E*)-β-Damascenone	1361	1361	-	-	t	0.3	3.6	t	O13
128	Methyl eugenol	1369	1369	0.1	-	-	-	-	-	Ar
129	α-Copaene	1375	1379	0.3	-	-	-	-	-	SH
130	*cis*-β-Elemene	1377	1381	-	-	0.1	-	-	-	SH
131	Dodecan-2-one	1385	1377	-	-	-	-	0.5	-	AO
132	Dodecanal	1385	1386	-	-	-	0.7	0.2	-	AO
133	β-Elemene	1386	1389	0.3	0.9	-	-	-	-	SH
134	(*E*)-β-Damascone	1392	1398	-	-	-	-	1.0	-	O13
135	7,8-Dihydro-β-damascenone	1396	1424	-	-	-	-	0.7	-	O13
136	Tetradecane	1400	1400	-	0.1	0.1	-	-	-	AH
137	(*E*)-α-Ionone	1404	1413	-	t	0.1	0.1	-	0.2	O13
138	α-Barbatene	1411	1414	-	0.9	-	-	-	-	SH
139	*cis*-α-Bergamotene	1412	1411	-	0.1	-	-	-	-	SH
140	α-Santalene	1416	1422	-	2.0	-	-	-	-	SH
141	Geranylacetone	1427	1428	0.1	0.6	0.1	0.6	2.7	0.7	O
142	*γ*-Elemene	1427	1429	0.5	-	-	-	-	-	SH
143	*trans*-α-Bergamotene	1431	1434	-	0.1	-	-	-	-	SH
144	Isobazzanene	1439	1442	-	0.5	-	-	-	-	SH
145	β-Barbatene	1444	1445	-	**5.3**	-	-	-	-	SH
146	Sesquisabinene B	1445	1445	0.6	-	-	-	-	-	SH
147	4-(2,4,4-Trimethyl-cyclohexa-1,5-dienyl)-but-3-en-2-one	1456	-	-	-	-	-	0.8	-	O13
148	β-Santalene	1458	1460	-	0.4	-	-	-	-	SH
149	β-Ionone epoxide	1459	1456	0.1	0.4	0.2	0.9	3.3	3.7	O13
150	(*E*)-β-Ionone	1462	1468	t	0.6	0.8	1.1	**5.7**	t	O13
151	4,5-di-*epi*-Aristolochene	1465	1470	-	0.1	-	-	-	-	SH
152	γ-Muurolene	1470	1474	0.5	-	-	-	-	-	SH
153	ar-Curcumene	1471	1473	-	0.2	-	-	-	-	SH
154	γ-Curcumene	1472	1475	-	0.1	-	-	-	-	SH
155	5-*epi*-Aristolochene	1473	1477	0.1	-	-	-	-	-	SH
156	Tridecan-2-one	1474	1476	-	0.6	-	0.2	-	-	AO
157	*trans*-β-Bergamotene	1477	1480	-	0.1	-	-	-	-	SH
158	3,4-Dimethyl-5-pentyl-5*H*-furan-2-one	1480	1481	-	1.1	0.1	-	2.4	1.5	O
159	Aristolochene	1481	1486	0.7	-	-	-	-	-	SH
160	Dihydroactinidiolide	1487	1487	-	-	-	-	0.8	-	O
161	Tridecanal	1488	1490	-	0.3	-	1.0	-	-	AO
162	α-Selinene	1490	1494	0.7	-	-	-	-	-	SH
163	Cuparene	1493	1498	-	1.3	-	-	-	-	SH
164	Pentadecane	1500	1500	-	-	-	-	0.3	-	AH
165	Germacrene A	1500	1503	-	-	0.7	-	-	-	SH
166	β-Bisabolene	1502	1503	-	0.2	-	-	-	-	SH
167	Methyl dodecanoate	1504	1507	-	-	-	-	0.7	-	AO
168	γ-Cadinene	1505	1507	0.2	-	0.3	0.3	-	-	SH
169	*trans*-Calamenene	1508	1517	0.1	-	-	-	-	-	SH
170	Photosantalol	1509	1511	-	0.2	-	-	-	-	SO
171	*δ*-Cadinene	1518	1520	0.7	0.1	-	t	-	-	SH
172	Selina-4(15),7(11)-diene	1528	1534	0.4	-	-	-	-	-	SH
173	Dodecanoic acid	1546	1554	-	-	-	1.3	4.1	-	AO
174	(*E*,*E*)-Pseudoionone	1555	1563	-	-	-	-	0.3	-	O13
175	(2*E*)-2-Methyl-4-(2,6,6-trimethylcyclohex-1-en-1-yl)but-2-enal	1568	1584	-	-	-	-	0.4	-	O13
176	Maaliol	1573	1565	-	0.5	-	-	-	-	SO
177	Globulol	1574	1578	-	-	-	0.6	1.2	1.7	SO
178	Tetradecanal	1592	1592	-	1.0	-	0.9	-	-	AO
179	Hexadecane	1600	1600	-	-	-	0.1	0.1	-	AH
180	Butylphthalide	1610	1616	0.4	-	-	-	-	-	Ar
181	ar-Bisabolol	1613	1619	-	0.1	-	-	-	-	SO
182	T-Cadinol	1624	1623	-	-	-	0.1	-	-	SO
183	T-Muurolol	1626	1633	0.2	-	-	-	-	-	SO
184	Vulgarone B	1630	1632	-	**14.9**	-	-	-	-	SO
185	α-Cadinol	1639	1642	-	-	-	0.2	-	-	SO
186	Pogostol	1639	1647	-	-	t	-	-	0.3	SO
187	(*Z*)-Butylidenphthalide	1641	1644	**8.5**	-	-	-	-	-	Ar
188	α-Barbatenal	1652	1659	-	0.5	-	-	-	-	SO
189	Hexahydrofarnesol	1659	1667	-	-	-	-	0.8	-	O
190	Tetradecanol	1664	1670	-	-	-	0.5	-	-	AO
191	*α*-Bisabolol	1670	1673	-	-	-	0.3	0.3	1.0	SO
192	Acorenone	1671	1681	-	4.0	-	-	-	-	SO
193	Pentadecan-2-one	1676	1677	-	t	-	0.2	0.2	-	AO
194	(*E*)-Butylidenphthalide	1681	1673	0.8	-	-	-	-	-	Ar
195	Pentadecanal	1692	1693	-	**5.8**	0.6	-	1.6	1.7	AO
196	Heptadecane	1700	1700	-	-	-	0.1	0.4	-	AH
197	(*Z*)-Ligustilide	1703	1732	**11.0**	-	-	-	-	-	Ar
198	Methyl myristate	1710	1713	-	-	-	0.1	0.5	-	AO
199	Phenanthrene	1746	1744	-	0.9	-	-	-	-	Ar
200	Myristic acid	1747	1748	0.3	-	-	0.9	3.3	-	AO
201	(*E*)-Ligustilide	1756	1782	0.1	-	-	-	-	-	Ar
202	Hexadecanal	1793	1782	-	0.1	-	0.3	0.2	-	AO
203	Octadecane	1800	1800	-	t	-	0.3	0.5	0.3	AH
204	Hexahydrofarnesyl acetone	1829	1832	1.0	-	0.4	-	**13.4**	0.3	O
205	Farnesylacetone	1894	1895	-	-	-	-	1.9	-	O
206	Nonadecane	1900	1900	-	0.2	-	0.5	0.5	0.7	AH
207	Methyl palmitate	1904	1904	0.2	0.3	0.1	0.7	1.8	3.0	AO
208	Isophytol	1939	1949	-	-	-	0.4	0.9	0.3	O
209	Palmitic acid	1945	1951	1.1	-	-	0.6	0.9	-	AO
210	Ethyl palmitate	1974	1954	-	-	-	-	-	0.3	AO
211	Eicosane	2000	2000	-	-	-	0.2	-	0.3	AH
212	Fluoranthene	2026	2020	-	0.9	-	-	-	-	Ar
213	Methyl linoleate	2067	2046	-	0.1	-	0.3	0.4	0.2	AO
214	Methyl palmitate	2071	2102	0.1	-	-	-	-	-	AO
215	Methyl linolenate	2072	2102	-	0.2	-	0.7	1.3	0.3	AO
216	Pyrene	2076	2070	-	0.6	-	-	-	-	Ar
217	Methyl oleate	2087	2082	-	-	-	-	-	0.2	AO
218	Heneicosane	2100	2100	-	0.3	-	0.3	0.3	0.2	AH
219	Phytol	2107	2104	0.5	0.3	0.1	0.2	2.8	0.1	O
220	Docosane	2200	2200	-	-	-	-	0.2	0.2	AH
221	Tricosane	2300	2300	0.3	0.4	0.1	0.6	1.8	1.4	AH
222	Tetracosane	2400	2400	-	0.1	-	-	0.2	0.2	AH
223	Pentacosane	2500	2500	0.5	0.3	0.1	0.4	1.7	1.7	AH
224	Hexacosane	2600	2600	-	0.1	-	-	-	-	AH
225	Heptacosane	2700	2700	0.2	-	-	-	-	-	AH
226	Nonacosane	2900	2900	0.5	-	-	-	-	-	AH
	Total identified constituents			**82.5**	**87.4**	**88.2**	**86.3**	**84.2**	**61.7**	
	Aliphatic hydrocarbons AH			1.5	1.5	0.3	2.7	6.0	5.0	
	Oxygenated aliphatics AO			7.2	20.0	42.8	26.1	23.3	24.0	
	Monoterpene hydrocarbons MH			9.9	0.1	0.1	0.4	-	0.1	
	Oxygenated monoterpenes MO			28.2	20.3	24.7	42.8	5.4	12.1	
	Sesquiterpene hydrocarbons SH			5.1	12.6	1.1	0.3	-	-	
	Oxygenated sesquiterpenes SO			0.2	20.2	-	1.2	1.5	3.0	
	Aromatic compounds Ar			23.0	8.1	14.4	7.8	5.7	9.3	
	Other O + O13			7.3 + 0.1	3.4 + 1.2	3.7 + 1.1	3.6 + 2.4	26.5 + 15.8	4.3 + 3.9	
	Identified compounds			76	94	70	88	80	54	
	Oil yield			0.22	0.19	0.24	0.16	0.10	0.14	

**Table 2:** Comparison of the antioxidant activity of *Impatiens* L. essential oils and standard antioxidants. Different superscripts in each column indicate significant differences in the means at *p* < 0.05.

Essential Oils	Radical Scavenging Activity DPPH (EC_50_, µg/mL)	Inhibition of Linoleic Acid Peroxidation (IC_50_, µg/mL)
*I. balsamina herb* (IBH)	16.14 ± 0.68 ^g^	468.06 ± 2.03 ^f^
*I. glandulifera herb* (IGH)	3.96 ± 0.03 ^b^	102.08 ± 0.71 ^b^
*I. glandulifera roots* (IGR)	5.84 ± 0.03 ^d^	116.98 ± 0.43 ^c^
*I. noli-tangere herb* (INH)	4.76 ± 0.05 ^b,c^	123.18 ± 1.34 ^c^
*I. parviflora herb* (IPH)	9.06 ± 0.07 ^e^	190.94 ± 0.76 ^d^
*I. parviflora roots* (IPR)	10.38 ± 0.17 ^f^	323.66 ± 0.24 ^e^
*Ascorbic acid*	2.05 ± 0.01 ^a^	-
*BHT*	-	18.21 ± 0.11 ^a^

Means values followed by different superscripts (a–g) in a column are significantly different (*p* < 0.05).
